# The molecular evolution of spiggin nesting glue in sticklebacks

**DOI:** 10.1111/mec.13317

**Published:** 2015-08-03

**Authors:** P. J. Seear, E. Rosato, W. P. Goodall‐Copestake, I. Barber

**Affiliations:** ^1^ Department of Neuroscience, Psychology and BehaviourCollege of Medicine, Biological Sciences and PsychologyUniversity of LeicesterLeicesterLE1 7RHUK; ^2^ Department of GeneticsCollege of Medicine, Biological Sciences and PsychologyUniversity of LeicesterLeicesterLE1 7RHUK; ^3^ British Antarctic SurveyHigh CrossMadingley RoadCambridgeCB3 0ETUK

**Keywords:** adaptation, *Gasterosteus aculeatus*, gene duplication, gene family, nest building, retrotransposon

## Abstract

Gene duplication and subsequent divergence can lead to the evolution of new functions and lineage‐specific traits. In sticklebacks, the successive duplication of a mucin gene (*MUC19*) into a tandemly arrayed, multigene family has enabled the production of copious amounts of ‘spiggin’, a secreted adhesive protein essential for nest construction. Here, we examine divergence between spiggin genes among three‐spined sticklebacks (*Gasterosteus aculeatus*) from ancestral marine and derived freshwater populations, and propose underpinning gene duplication mechanisms. Sanger sequencing revealed substantial diversity among spiggin transcripts, including alternatively spliced variants and interchromosomal spiggin chimeric genes. Comparative analysis of the sequenced transcripts and all other spiggin genes in the public domain support the presence of three main spiggin lineages (*spiggin A*,* spiggin B* and *spiggin C*) with further subdivisions within *spiggin B* (*B1*,* B2*) and *spiggin C* (*C1*,* C2*). *Spiggin A* had diverged least from the ancestral *MUC19*, while the *spiggin C* duplicates had diversified most substantially. In silico translations of the spiggin gene open reading frames predicted that spiggins A and B are secreted as long mucin‐like polymers, while spiggins C1 and C2 are secreted as short monomers, with putative antimicrobial properties. We propose that diversification of duplicated spiggin genes has facilitated local adaptation of spiggin to a range of aquatic habitats.

## Introduction

Gene duplication can lead to an increase in protein product and – following the divergence of duplicated genes – novel protein functions (Ohno 1970; Lynch & Force 2000) and lineage‐specific traits (Wu *et al*. 2009; Vonk *et al*. 2013). Studies of bacteria (Hastings *et al*. 2000; Riehle *et al*. 2001), protists (Kaufmann & Klein 1992; Reinbothe *et al*. 1993), fungi (Tohoyama *et al*. 1996; Brown *et al*. 1998), plants (van Hoof *et al*. 2001; Widholm *et al*. 2001) and invertebrates (Otto *et al*. 1986; Lenormand *et al*. 1998) have demonstrated that gene duplication can play a significant role in adaptive evolution. In primates, the accelerated expansion of several gene families also suggests evidence for adaptive evolution (Hahn *et al*. 2007). In a study of recently duplicated genes in bacteria, archaea and eukaryotes, Kondrashov *et al*. (2002) found that most of these genes were involved in environmental interactions, with a significant proportion encoding membrane or secreted proteins. Consequently, gene duplication has been suggested as a general mechanism promoting adaptation to novel environmental conditions (Kondrashov 2012).

In sticklebacks, a multigene family (Jones *et al*. 2001; Kawasaki *et al*. 2003; Kawahara & Nishida 2006, 2007) is known to encode the protein component of a glue (‘spiggin’) that is produced in the kidney of males and used in the construction of nests (Wootton 1976; Jakobsson *et al*. 1999). Building an effective nest is essential for successful reproduction in sticklebacks, as the nest not only protects the eggs and developing fry, but also serves as a focus for courtship (Barber *et al*. 2001; Östlund‐Nilsson & Holmlund 2003). The ancestral spiggin gene is thought to have originated from the duplication of the single‐copy vertebrate mucin gene, *MUC19*, with duplication occurring both before and after the divergence of three‐spined (*Gasterosteus aculeatus*) and nine‐spined (*Pungitius pungitius*) sticklebacks (Kawahara & Nishida 2007). Three‐spined sticklebacks have colonized a wide range of ecologically diverse freshwater aquatic habitats from ancestral marine refugia (Bell & Foster 1994; Jones *et al*. 2012) and hence provide an ideal opportunity to examine the role of gene duplication in the adaptive evolution of ecologically important lineage‐specific traits.

The initial duplications of spiggin might simply have involved the addition of functionally equivalent genes due to a beneficial increase in gene dosage (Kondrashov *et al*. 2002), possibly because this would lead to glue being synthesized in greater quantities, or at a faster rate. Analysis of the spiggin multigene family by Kawahara & Nishida (2007), however, revealed three subgroups of genes, indicating that the duplicated genes have subsequently undergone divergence. Because sticklebacks have colonized a wide variety of freshwater habitats, the glue – which is secreted by nesting males directly into the external environment – must function across a wide variety of ecological conditions, giving the potential for population‐level adaptation of the glue to local physicochemical conditions. It is currently not known which suites of diverged genes are present in the genomes of different stickleback populations.

In this study, we provide substantial further characterization of the spiggin multigene family by Sanger sequencing spiggin transcripts from northern European marine and freshwater three‐spined sticklebacks, and also from a freshwater population of nine‐spined sticklebacks. In addition to revealing spiggin gene differences between stickleback populations, this approach identified four novel interchromosomal chimeric spiggin genes from *G. aculeatus* and two novel interchromosomal chimeric spiggin genes from *P. pungitius*. Comparative analyses of these and other spiggin genes from the public domain resolved three major spiggin lineages, with further duplications evident within these lineages. Analysis of in silico translations of the sequenced genes revealed significant differences in the number and location of glycosylation sites and multimerisation motifs, in addition to overall protein length, strongly suggesting different functional properties between spiggin proteins from different spiggin gene lineages. Finally, we discuss different gene duplication mechanisms and provide evidence for retrotransposon involvement in the amplification of the spiggin multigene family.

## Materials and methods

### Fish sampling, husbandry and dissection

Adult freshwater *Gasterosteus aculeatus* were collected from a pond in Inverleith Park, Edinburgh, UK (‘Edinburgh’: 55°57′41.24″N, 3°13′4.76″W). Adult freshwater *Pungitius pungitius* were sampled from the River Welland in Leicestershire, UK (‘Welland’: 52°28′33.76″N, 0°55′22.00″W). All sticklebacks were collected using wire mesh minnow traps in April 2012. Fish were transported to aquarium facilities at the University of Leicester and maintained under static conditions in 70‐L holding tanks, under controlled temperature (20 °C) and a 14 h:10 h light:dark photoperiod. Fish were fed daily ad libitum on frozen *Chironomus* sp. larvae throughout.

Adult marine *G. aculeatus* were caught by seine net from the Gullmarsfjord at Sälvik on the island of Skaftö, off the west coast of Sweden (‘Gullmarsfjord’: 58°14′33.76″N, 11°28′7.41″E) in May 2012. The fish were caught during their migration from the main channel of the fjord to the nesting grounds, which are shallow sandy beaches in the inner fjord. Salinity at the site of capture was 19.8‰, which is typical of surface water salinity during that time of year. Fish were transferred to the Sven Lovén Centre for Marine Sciences at Fiskebäckskil and maintained in 72‐L holding tanks provided with a flow of temperature‐controlled (15 °C) surface water, pumped from 5 m depth in the Gullmarsfjord. Fish were fed daily ad libitum on frozen adult brine shrimp *Artemia* sp. throughout the study, and day length was controlled to simulate natural regimes during the breeding season at this northerly latitude (19 h:5 h light:dark).

As male sticklebacks from each population developed nuptial coloration and showed signs of initiating nesting behaviour, they were removed from the holding tanks and transferred individually to nesting tanks. Nesting tanks were provided with a substratum of washed sand (3 cm depth) and plastic plants for cover. These tanks were additionally supplied with nesting material (150, 5‐cm‐long polyester threads and (for marine fish) a clump of brown filamentous algae). Males in the nesting tanks were enticed daily with a free‐swimming female for 20 min to stimulate nesting behaviour and checked daily for signs of nest building.

Once a nest had been constructed, the male was euthanized using U.K. Home Office approved Schedule 1 techniques (Benzocaine‐induced deep anaesthesia followed by spinal cord severance). The kidney, which is the organ in which spiggin is synthesized, was immediately removed, and placed in RNA*later* ^®^ solution (Life Technologies, Carlsbad, CA, USA) for subsequent analysis of spiggin gene expression. A pectoral fin sample was also taken from each fish *post mortem*, and preserved in ethanol for subsequent DNA extraction.

### Cloning and sequencing of spiggin transcripts

Kidney samples were taken from three freshwater (Edinburgh) and three marine (Gullmarsfjord) *G. aculeatus* individuals, and from three freshwater (Welland) *P. pungitius* individuals. Total RNA was extracted from RNA*later* ^®^‐preserved kidneys of male sticklebacks using the RNeasy Plus Mini kit (Qiagen GmbH, Hilden, Germany) following the manufacturer's instructions. RNA was eluted into DEPC‐treated water and the concentration and purity determined using a NanoDrop spectrophotometer (LabTech International, Lewes, UK). One microgram of total RNA was electrophoresed on a nondenaturing 1.5% (w/v) agarose gel to check for degradation. The 5′ ends of spiggin genes were amplified by RACE‐PCR from three micrograms of total RNA using a GeneRacer Kit (Life Technologies) following the manufacturer's instructions. Touchdown PCR cycling conditions were as follows: 94 °C for 2 min, followed by 5 cycles of 94 °C for 30 s, 72 °C for 1.5 min, 5 cycles of 94 °C for 30 s, 70 °C for 1.5 min and 25 cycles of 94 °C for 30 s, 65 °C for 30 s, 72 °C for 1.5 min, with a final extension of 72 °C for 10 min. The internal primers SPG5R01, SPG5R04 as used by Kawahara & Nishida (2006) and a new primer SPG5A1C1 (Table S1, Supporting information), based upon partial spiggin gene transcript sequences (GenBank: JK993477‐JK993535; Seear *et al*. 2014), were used for generic spiggin gene family PCR amplification. PCR products were electrophoresed on a 1.5% (w/v) agarose gel and excised before purifying with a MinElute Gel Extraction Kit (Qiagen). Purified PCR products were then cloned into pCR^®^4‐TOPO vector using a TOPO^®^ TA Cloning for Sequencing Kit (Life Technologies) following the manufacturer's instructions. A total of 52 clones were isolated from overnight LB cultures using an ISOLATE Plasmid Mini Kit (Bioline, London, UK) prior to sequencing with universal M13F and M13R primers by Genome Enterprise Limited (Norwich, UK). Nucleotide sequences of the partial spiggin cDNAs were processed in Geneious^®^ 6.1.6 (Biomatters: http://www.geneious.com) to remove vector and low‐quality sequence before using blastn (Altschul *et al*. 1997) to search the NCBI nonredundant (nr) database for confirmation that the obtained cDNAs were *G. aculeatus* or *P. pungitius* spiggin gene produ‐cts. Spiggin gene transcripts were amplified from the *G. aculeatus* and *P. pungitius* GeneRacer cDNAs using the GeneRacer 3′ primer (0.6 μm) provided in the kit and the Spg5F1 primer (0.2 μm) that was conserved against all 5′ ends sequenced (Table S1, Supporting information). Touchdown PCR cycling conditions were as follows: 94 °C for 2 min, followed by 5 cycles of 94 °C for 30 s, 72 °C for 6 min, 5 cycles of 94 °C for 30 s, 70 °C for 6 min and 25 cycles of 94 °C for 30 s, 68 °C for 30 s, 72 °C for 6 min, with a final extension of 72 °C for 10 min. The amplified products were cloned into pCR‐XL‐TOPO^®^ vector using a TOPO^®^ XL PCR Cloning Kit following the manufacturer's instructions, and 48 clones from each individual were isolated and sequenced as above. For clones longer than 1.6 kb in length, primer walking was used to sequence the entire length of the transcript (Table S1, Supporting information). Nucleotide sequences were processed using Geneious^®^ 6.1.6 as above.

### DNA characterization and alignment

The Ensembl Genome Browser (http://www.ensembl.org/index.html) and the UC‐Santa Cruz Genome Browser (http://sticklebrowser.stanford.edu/cgi-bin/hgGateway) were used to search the February 2006 draft (Broad/gasAcu1) assembly of *G. aculeatus* (Jones *et al*. 2012) for the location of all sequenced spiggin transcripts using the blat search tool (Kent 2002). The *Gasterosteus aculeatus* – WGS database in the NCBI trace archive and the SRA *Gasterosteus aculeatus* (WGS) database in the NCBI sequence read archive, along with the NCBI EST, nucleotide and genomic survey sequence (gss) databases were also used to search for transcripts that did not match the reference assembly.

Unique *G. aculeatus* sequences generated in this study (*n* = 73) were aligned with 21 sequences from the public domain using Geneious^®^ 6.1.6 with default settings (65% similarity matrix and gap opening and extension penalties of 12 and 3, respectively). These additional sequences included all published spiggin cDNAs (Jones *et al*. 2001; Kawasaki *et al*. 2003; Kawahara & Nishida 2006, 2007), representatives from each of the six Ensembl spiggin gene predictions from the 2006 draft assembly of *G. aculeatus*, and a single *MUC19* gene from the cichlid fish, *Neolamprologus brichardi* as the out‐group (Table S2, Supporting information). To identify major spiggin phylogenetic lineages and to characterize which DNA positions corresponded with each lineage, we analysed the alignment using two complimentary methods: (i) manual inspection of parsimony‐informative site patterns along the entire 94 sequence alignment; and (ii) sliding‐window phylogenetic analyses of alternative six‐taxon alignment partitions, conducted using the Geneious Dual Brothers recombination detection plugin with default settings for an analysis of all possible topologies (Minin *et al*. 2005).

Open reading frames (ORFs) of sequenced spiggin transcripts were predicted using the ncbi orf finder (http://www.ncbi.nlm.nih.gov/projects/gorf/), and following in silico translation, protein domains were identified using the NCBI conserved domain search against the conserved domain database v3.10 (Marchler‐Bauer *et al*. 2011). Signal peptide cleavage sites and O‐linked and N‐linked glycosylation sites were predicted using signalp 4.1 (Petersen *et al*. 2011), netoglyc 4.0 (Steentoft *et al*. 2013) and netnglyc 1.0, respectively, in the CBS prediction servers (http://www.cbs.dtu.dk/services). The RepeatMasker table available from the UCSC genome browser (www.genome.ucsc.edu) was used to search the 2006 draft (Broad/gasAcu1) assembly of *G. aculeatus* for annotated Long Interspersed Nuclear Element‐1 (LINE‐1/L1) retrotransposons.

### Cloning and sequencing of chimeric genes from genomic DNA

Genomic DNA was extracted from fin clips of a marine (Gullmarsfjord) and a freshwater (Edinburgh) *G. aculeatus* using a DNeasy Blood and Tissue Kit (Qiagen). The *spiggin B/ChrIX* interchromosomal chimeric gene was amplified from this DNA by PCR using Platinum^®^ *Taq* DNA Polymerase High Fidelity (Life Technologies) with 0.2 μm of SPG5F1 and ChrIX R primers (Table S1, Supporting information). PCR conditions were as follows: 94 °C for 2 min, followed by 35 cycles of 94 °C for 30 s, 60 °C for 30 s and 68 °C for 1 min, with a final extension of 68 °C for 5 min. PCR products were electrophoresed on a 2% (w/v) agarose gel, and single bands of the expected size were excised and purified with a MinElute Gel Extraction Kit (Qiagen). Purified PCR products were cloned into pCR^®^4‐TOPO vector using a TOPO^®^ TA Cloning for Sequencing Kit (Life Technologies) following the manufacturer's instructions. Clones were isolated, sequenced and analysed as above. Genomic DNA was also extracted from fin clips of UK freshwater *G. aculeatus* sourced from Carsington Reservoir in Derbyshire, UK (53°03′52.35″N, 1°38′30.94″W), and the River Welland in Leicestershire, UK, to partially clone and sequence the *spiggin B/C1* and *spiggin B/C2* intrachromosomal chimeric genes, using the methods described above. Both genes were amplified from this genomic DNA by PCR using RedTaq ReadyMix (Sigma) with 0.5 μm of Spg alpha F and Spg alpha1R primers (Table S1, Supporting information). PCR conditions were as follows: 94 °C for 2 min, followed by 35 cycles of 94 °C for 30 s, 63 °C for 30 s and 72 °C for 1 min, with a final extension of 72 °C for 10 min. PCR products were isolated, cloned and sequenced as above (Table S3, Supporting information). The *spiggin B/C1* and *spiggin B/C2* intrachromosomal chimerics were verified using additional internal reverse primers Spg C1 R3 and Spg C2 R2 (Table S1, Supporting information), respectively (Fig. S1, Supporting information), with the following PCR conditions: 94 °C for 2.5 min, followed by 35 cycles of 94 °C for 30 s, 62 °C (*spiggin B/C1*)/65 °C (*spiggin B/C2*) for 30 s and 72 °C for 1 min, with a final extension of 72 °C for 5 min. PCRs were performed in duplicate.

## Results

### Cloning and sequencing of spiggin genes

From the three freshwater (Edinburgh) and three marine (Gullmarsfjord) *Gasterosteus aculeatus* fish, 237 clones partially sequenced with M13 forward and reverse primers were identified as spiggin transcripts thorough blastn searches (*E* value <1e^−18^). A total of 84 clones were sequenced in full. These clones included all alternatively spliced and the most divergent forms of spiggin transcript, including both intra‐ and interchromosomal chimeric spiggin sequences. blat searches of the 2006 draft (Broad/gasAcu1) assembly of *G. aculeatus*, in addition to blast searches of the NCBI trace archives and nucleotide, EST and SRA databases revealed that 73 of these transcripts were unique (Table S2, Supporting information). From *Pungitius pungitius*, a total of 127 partially sequenced clones showed significant sequence similarity (*E* value <1e^−18^) to published spiggin sequences, including two interchromosomal chimeric transcripts identified through blat searches of the *G. aculeatus* draft assembly.

### Spiggin gene alignments, characterization and phylogeny

**Figure 1:**
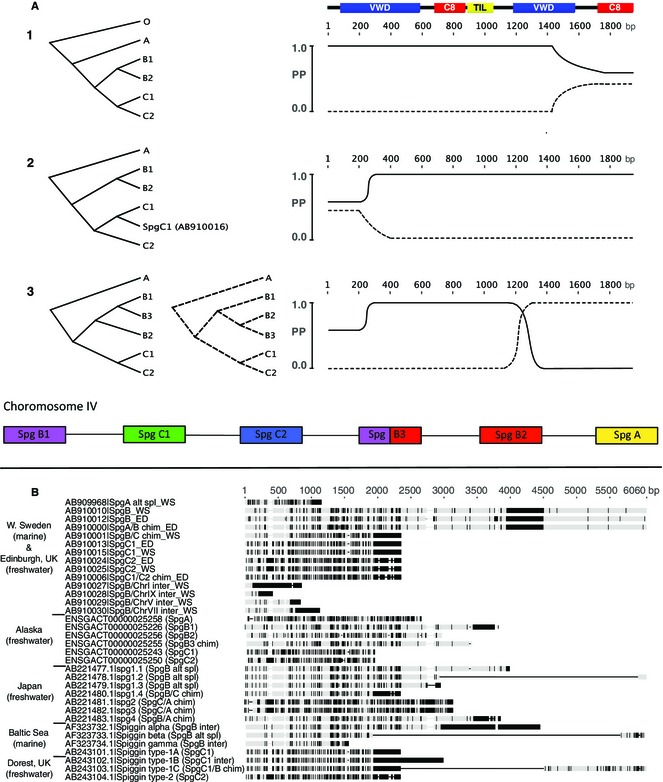
Spiggin gene phylogenies and alignments. (A) Results from sliding‐window phylogenetic analysis of five *Gasterosteus aculeatus* draft genome spiggin transcript predictions annotated as A, B1, B2, C1 and C2. In profile 1, these transcripts were rooted with a *MUC19* out‐group (O). Profiles 2 and 3 were rooted using *spiggin A* and show the impact of adding nonchimeric *spiggin C1* (*SpgC1*, AB910016 from this study) and chimeric *spiggin B3* (*G. aculeatus* draft genome) sequences, respectively. Posterior probabilities of the two most frequent topologies along the alignment are indicated by solid and dashed lines. Positions along the alignment in base pairs (bp) are set below a generalized in silico spiggin translation with the protein domains: von Willebrand factor type D domain, C8 domain and trypsin inhibitor‐like cysteine‐rich domain. A simplified view of the tandemly arrayed spiggin genes on chromosome IV of the stickleback draft genome is shown below the phylogenies. (B) Nucleotide alignment of a representative selection of spiggin transcripts sequenced in this study (West Sweden and Edinburgh, UK), along with all published spiggin sequences and the six spiggin transcript predictions from the draft genome assembly of *G. aculeatus* (Alaska) used in the sliding‐window phylogenies (A). Light grey indicates consensus between sequences, and black indicates nucleotide differences. WS, West Sweden; ED, Edinburgh; alt spl, alternatively spliced; chim, intrachromosomal chimeric; inter, interchromosomal chimeric. All spiggin transcripts have been further annotated with the spiggin A, B and C nomenclature.

The 73 unique *G. aculeatus* spiggin recombinant clone sequences were aligned with 20 *G. aculeatus* spiggin sequences from the public domain and a single *MUC19* gene as the out‐group (Table S2, Supporting information). Sliding‐window phylogenetic analysis resolved three major spiggin lineages (A, B and C) and further subdivisions within two of these lineages (B, C) (Fig. 1A). The use of an out‐group revealed that spiggin lineage A diverged first, followed by the split of lineages B and C. However, this simple scenario was complicated by the fact that different regions of the alignment supported different topologies. By characterizing the nucleotide site patterns and blastn matches to these regions, we found that 14 of the transcripts from this study, along with eight published *G. aculeatus* spiggin cDNA sequences and one of the six spiggin loci from the *G. aculeatus* draft genome assembly, were chimeric. The chimeric transcripts included different combinations of the five spiggin gene lineages (intrachromosomal chimerics) and also a mix of spiggin with other gene sequences (interchromosomal chimerics) (Fig. 1, Table S2, Supporting information). The presence of chimeric sequences, coupled with regions of low phylogenetic signal, meant that it was not always possible to characterize all sites within the alignment into the subdivisions B1 or B2. For this reason, we simply refer to type ‘B’ lineages when an absence of data prevented more specific lineage identifications.

### Spiggin chimeric transcripts identified from sequencing in this study

**Figure 2:**
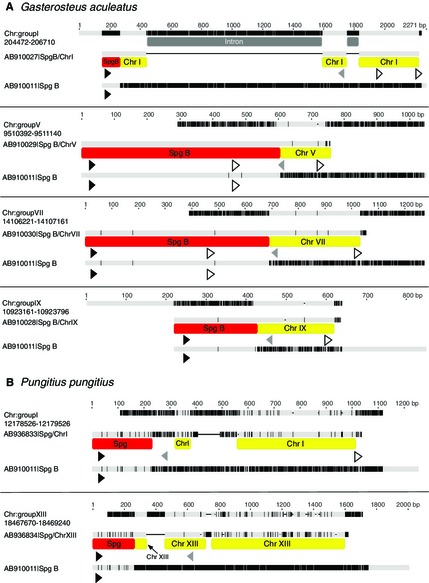
Nucleotide alignments of *Gasterosteus aculeatus* and *Pungitius pungitius* interchromosomal spiggin chimerics with the 2006 draft (Broad/gasAcu1) assembly of *G. aculeatus* and parental genes. Alignments of the *G. aculeatus* (A) and *P. pungitius* (B) interchromosomal spiggin chimerics with *spiggin B* (AB910011) and the recruited region of the draft assembly. The spiggin region of the *P. pungitius* interchromosomal chimeric has not been annotated with a specific spiggin gene as the spiggin multigene family in *P. pungitius* has not been fully characterized. Putatively assigned introns are indicated in grey. Black indicates nucleotide differences between sequences within the alignment. Black triangles represent start codons, grey triangles represent stop codons, and the white triangles indicate polyadenylation signals (AAUAAA). Chr, chromosome.

**Figure 3:**
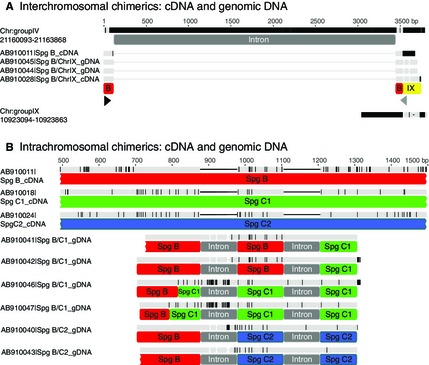
Genomic and cDNA alignments of spiggin chimerics. (A) Nucleotide alignments of cDNA and genomic DNA of the *spiggin B/ChrIX* interchromosomal spiggin chimeric with the recruited regions of chromosomes IV and IX and *spiggin B* (AB910011). Black and grey triangles indicate start and stop codons, respectively. (B) Nucleotide alignments of intrachromosomal spiggin chimerics with parental genes, *spiggin B*,* spiggin C1* and *spiggin C2*. Black indicates nucleotide differences between each sequence and the consensus sequence. Putatively assigned introns and alignments with parental genes are annotated below each chimeric gene. B, *Spiggin B*; IX, chromosome IX.

The 14 *G. aculeatus* chimeric spiggin transcripts sequenced in this study comprised ten intrachromosomal chimerics and four interchromosomal chimerics. Interchromosomal chimeric transcripts consisted of variable lengths of *spiggin B* at the 5′ end (which does not always include intact exons), fused to variable lengths of sequence from chromosomes I, V, VII or IX at the 3′ end (Fig. 2A and Table S2, Supporting information). blat searches of the *G. aculeatus* draft genome assembly revealed that the nonspiggin recruited regions of *spiggin B/ChrI* and *spiggin B/ChrV* interchromosomal chimeric transcripts corresponded to eukaryotic translation initiation factor 4 h (*eif4h*) and sulfotransferase family 1 cytosolic sulfotransferase 6 (*sult1st6*), respectively (Table S2, Supporting information). No introns were observed in the genomic DNA sequences of the *spiggin B/ChrIX* gene from marine (Gullmarsfjord) and freshwater (Edinburgh) three‐spined sticklebacks (Figs 3A and S2, Supporting information). Two interchromosomal chimeric transcripts were also identified in the *P. pungitius* sequencing through blat searching of the *G. aculeatus* draft genome. These chimerics consisted of 230–266 bp of spiggin fused to 641–1108 bp of DNA from chromosomes I and XIII (Fig. 2B, Table S2, Supporting information).

The ten intrachromosomal chimeric transcripts sequenced in this study from *G. aculeatus* each consisted of various lengths of nucleotide sequence from two different spiggin genes (Table S2, Supporting information). *Spiggin B/C1* and *spiggin B/C2* chimeric genes were also successfully PCR amplified and partially sequenced from *G. aculeatus* genomic DNA, revealing the presence of introns (Fig. 3B). These intrachromosomal chimeric spiggin genes were further verified by PCR using additional reverse primers designed to span the chimeric breakpoint (Fig. S1, Supporting information).

### Spiggin chimeric transcripts identified from the public domain

**Figure 4:**
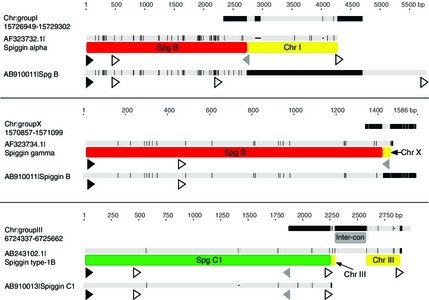
Nucleotide alignments of *Gasterosteus aculeatus* interchromosomal spiggin chimerics identified from previously published spiggin genes. Black indicates nucleotide differences between each sequence and the consensus sequence. Black triangles represent start codons, grey triangles represent stop codons, and the white triangles indicate polyadenylation signals (AAUAAA). Chr, chromosome; Inter‐con, inter‐contig (on the draft stickleback genome assembly).

*Spiggin alpha*,* spiggin gamma* (Jones *et al*. 2001) and *spiggin type‐1B* (Kawasaki *et al*. 2003) were identified as interchromosomal chimeric transcripts (Fig. 4 and Table S2, Supporting information). *Spiggin alpha* and *spiggin gamma* were similar to the interchromosomal chimerics identified from the sequencing in this study in that the 5’ end of the transcript consisted of *spiggin B*. With *spiggin type‐1B* however, the 5’ spiggin portion of the transcript consisted of *spiggin C1*. There were no interchromosomal spiggin genes observed in the 2006 draft (Broad/gasAcu1) assembly of *G. aculeatus*.

Five intrachromosomal spiggin chimerics were identified from the published spiggin genes. These were *spiggin type‐1C* (Kawasaki *et al*. 2003), and *spg1.4*,* spg 2*,* spg 3* and *spg 4* (Kawahara & Nishida 2006) (Table S2, Supporting information). From the draft genome assembly of *G. aculeatus*, one of the six spiggin loci (annotated on the assembly as spg4/ENSGACT00000025255, but revised in this study to *spiggin B3*) was identified as an intrachromosomal chimeric consisting of sequence from both *spiggin B1* (ENSGACT00000025226) and *spiggin B2* (ENSGACT00000025256).

### Presence of LINE‐1s in the *G. aculeatus* draft genome assembly

**Figure 5:**
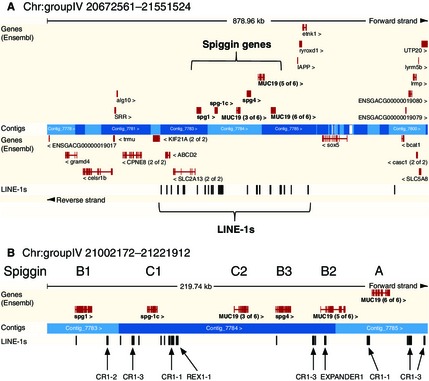
The spiggin multigene family as annotated on the 2006 draft (Broad/gasAcu1) assembly of *Gasterosteus aculeatus*. RepeatMasker has been used to highlight Long Interspersed Nuclear Element‐1s (LINE‐1s) for Chr:groupIV 20672561–21551524 (A) and Chr:groupIV 21002172–21221912 (B). The spiggin transcript nomenclature as revised in this study is shown above each spiggin gene (B). Figure is adapted from Ensembl Genome Browser data. CR1, Chicken repeat 1; REX1, retrotransposable elements first described in *X* *iphophorus* fish genome.

Analysis of an 878‐kb region of chromosome IV from the *G. aculeatus* draft assembly (Chr:group IV 20672561–21551524), which contains all annotated spiggin genes, revealed a cluster of 25 partial LINE‐1 sequences (Fig. 5A). The closest similar cluster of LINE‐1 sequences are located 1.5–2.0 Mb downstream of this spiggin gene region. Examination of the 219‐kb region of chromosome IV (Chr:groupIV 21002172–21221912) containing only spiggin genes revealed 10 partial LINE‐1 sequences that, originally 5–7 kb (Vandergon & Reitman 1994), have been truncated at the 5’ end to between 439 bp and 1512 bp in length (Fig. 5B). There is 99% nucleotide homology between two of the Chicken repeat 1‐3 (CR1‐3) LINE‐1s and 92% homology between 3 of the CR1‐1 LINE‐1s (Fig. 5).

### Spiggin protein predictions

**Figure 6:**
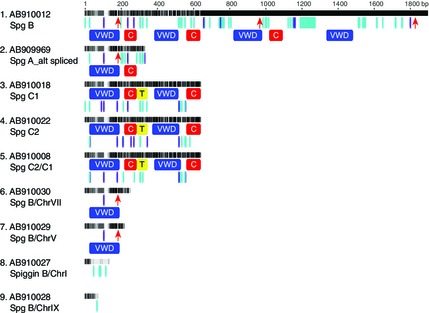
Alignments of in silico translations of spiggin transcript putative open reading frames. Below each translation are annotated predicted O‐linked glycosylation sites (light blue), N‐linked glycosylation sites (purple) and dimerization/multimerization motifs (red arrows). Coloured boxes in the protein alignments represent the following domains: vWD, von Willebrand factor type D domain (blue); C, C8 domain (red); T, trypsin inhibitor‐like cysteine‐rich domain (yellow). Light grey indicates amino acid differences between each of the translations.

To investigate whether the proteins putatively translated from the *spiggin* mRNAs sequenced in this study function as expected for a secreted glue‐like protein, in silico translations followed by conserved domain searches were performed on predicted open reading frames (ORFs) of both chimeric and nonchimeric transcripts. Signal peptides were predicted at the 5’ end of all spiggin‐putative ORFs, indicating that the mRNAs encode for secretary proteins. In silico translation of *G. aculeatus spiggin B‐*,* G. aculeatus spiggin B C1‐* and *G. aculeatus spiggin B C2*‐putative ORFs sequenced in this study revealed that spiggin B, at 1852–1869 amino acids in length, is almost three times as long as spiggin C1 (616 aa) and C2 (639 aa). Conserved domain searches identified four von Willebrand factor type D (vWD) domains and three cysteine‐rich (C8) domains in spiggin B, and two vWD domains, two C8 domains and one trypsin inhibitor‐like cysteine‐rich (TIL) domain in spiggins C1 and C2 (Fig. 6, translations 1, 3 and 4). Spiggin B has a number of similarities with other mucins, such as a CXGEC motif at the C‐terminal end of the protein (Fig. 6, translation 1) that has been shown to be required for dimerization of mucin monomers in the endoplasmic reticulum (Perez‐Vilar & Hill 1998). Spiggin B also has a CGLCG motif in vWD domains 1 and 3 (Fig. 6, translation 1), which is required for multimerization of mucin dimers in the trans‐Golgi compartments (Perez‐Vilar & Hill 1998). Spiggins C1 and C2 do not have the dimerization (CXGEC) motif and possess only a truncated GLCG motif in both vWD domains (Fig. 6, translations 3 and 4).

The spiggin B proteins are predicted to have between 84 and 99 O‐linked glycosylation sites that are clustered into regions outside of the vWD and C8 domains (Fig. 6, translation 1), in contrast to spiggins C1 and C2 that only have between 10 and 13 sites (Fig. 6, translations 3 and 4). The six *spiggin A* transcripts identified in this study were relatively short (133–1087 bp), suggesting they may have arisen through alternative splicing of a longer transcript. In silico translations of the putative ORF of the longest *spiggin A* transcript (312 aa) identified a relatively high number of nine O‐linked glycosylation sites, in addition to a multimerization motif in the first vWD domain (Fig. 6, translation 2).

In silico translations of the putative ORFs for the novel recombined *G. aculeatus* intrachromosomal chimerics sequenced herein showed partial or whole protein domain swaps between parental spiggin proteins, but with no difference in the order of protein domains (e.g. Fig. 6, translation 5). However, the predicted number and location of N‐ and O‐linked glycosylation sites did differ between spiggin chimerics and parental proteins (Fig. 6, translations 3–5). Translation of the putative ORF for each of the four *G. aculeatus* novel interchromosomal chimeric transcripts (Fig. 6, translations 6–9) revealed that two of the four chimeric proteins contained one vWD binding domain and a full multimerisation motif, but no predicted O‐linked glycosylation sites (Fig. 6, translations 6 and 7).

## Discussion

### Spiggin multigene family characterization

Comparative analysis of 73 *Gasterosteus aculeatus* spiggin gene transcripts sequenced in this study, together with 20 *G. aculeatus* spiggin sequences from the public domain, resolved three major spiggin lineages (A, B and C) with further subdivisions of lineages B (B1, B2) and C (C1, C2), which were consistent with a previous phylogenetic hypothesis for the presence of five spiggin gene copies (Kawahara & Nishida 2007). Of the three main spiggin lineages, *spiggin A* has diverged the least from the ancestral *MUC19* gene, while *spiggin B* and *spiggin C* have undergone further gene duplications, with *spiggin C* duplicates having diversified most substantially. Different suites of chimeric and nonchimeric spiggin sequences were found in separate populations of *G. aculeatus* from Alaska (Jones *et al*. 2012), Japan (Kawahara & Nishida 2006), Sweden (Jones *et al*. 2001; and this study) and UK (Kawasaki *et al*. 2003; and this study) (Fig. 1B). Although such differences may reflect relatively small sample sizes, they are also consistent with the hypothesis that population‐specific differences continually evolve within the spiggin multigene family (Kawahara & Nishida 2007), resulting in the presence of spiggin gene duplicates and chimerics that are unique to geographically and possibly ecologically distinct *G. aculeatus* populations.

### Chimeric spiggin transcripts

Fourteen *G. aculeatus* chimeric spiggin transcripts were sequenced in this study, including ten intrachromosomal chimerics and four interchromosomal chimerics. Generation of chimeric transcripts can occur as a result of PCR artefacts (Brakenhoff *et al*. 1991), or during mRNA transcription, either as a result of *trans*‐splicing of pre‐mRNAs (Gingeras 2009) or by combining two adjacent genes through intergenic splicing of mRNA (Akiva *et al*. 2005). Alternatively, chimeric transcripts can result from changes in the genome sequence. The sequencing of one complete interchromosomal chimeric gene from the genomic DNA of a freshwater and a marine *G. aculeatus* that lacked a 3.5‐kb intron in the *spiggin B* region of the gene (an indicator of retrotransposition), along with the sequencing of three partial intrachromosomal chimeric genes from the genomic DNA of two freshwater *G. aculeatus* (Fig. 3 and Table S3, Supporting information), provides evidence that the chimeric transcripts we identified have arisen through alterations in the genomic DNA. The hypothesis that spiggin chimerics have arisen through changes in the genome is further supported by the identification of an intrachromosomal chimeric spiggin gene in the draft assembly of the *G. aculeatus* genome (Fig. 1A) and the absence of homologous overlap regions in the interchromosomal chimerics (Figs 2, 3A and 4).

The two types of chimeric transcripts we have discovered may have been generated by a number of mechanisms. These include LINE‐1 (L1)‐mediated retrotransposition (a cause of gene copy number variation; Schrider *et al*. 2011, 2013), unequal crossing over events (which have been shown to underlie tandem duplication; Lu *et al*. 2012) and gene conversion (Chen *et al*. 2007). The *G. aculeatus* and *Pungitius pungitius* interchromosomal chimeric transcripts sequenced in this study, along with published spiggin genes – *spiggin alpha*,* spiggin gamma* (Jones *et al*. 2001) and *spiggin type‐1B* (Kawasaki *et al*. 2003) – all consist of variable amounts of *spiggin B* or *spiggin C1* fused to nonspiggin sequence, which for *G. aculeatus* is located on a different chromosome. These events are likely to have occurred by L1 retrotransposition of random lengths of *spiggin B* and *spiggin C1* from the spiggin multigene family on chromosome IV to a new position in the genome. The L1‐mediated insertion of *spiggin B* into *eif4h* and *sult1st6* to create the *spiggin B/ChrI* and *spiggin B/ChrV* interchromosomal chimerics would likely have destroyed the original genes, but with nonlethal effects, possibly due to both of these genes being members of large multigene families.

Full‐length L1 elements encode an RNA‐binding protein (ORF1) and a multifunctional protein (ORF2) with reverse transcriptase and endonuclease activities (Finnegan 2012). Following transcription of a full‐length L1 mRNA using its internal promoter, *ORF1* and *ORF2* are translated, and due to *cis* preference, specifically act on their encoding mRNA (Wei *et al*. 2001). The L1 mRNA is then reverse transcribed by the L1‐encoded reverse transcriptase, priming at nicks in the genomic DNA generated by the *ORF2*‐encoded endonuclease. Active L1 retrotransposons may also transfer their 3′ flanking DNA to a new genomic location, as L1 has a weak transcription termination signal that may be skipped in favour of a polyadenylation site downstream of the L1 (Moran *et al*. 1999; Goodier *et al*. 2000). A recent study of non‐long terminal repeat (non‐LTR) retrotransposons in the *G. aculeatus* genome identified nine full‐length L1s of the Tx1 clade with very high levels of similarity (Blass *et al*. 2012). The low copy number of these elements (of the order 10^2^) suggests they could represent active retrotransposons (Sassaman *et al*. 1997). Another feature of L1 retrotransposition is the loss of introns (Rogers 1985), and this was observed in the genomic DNA sequence of the *spiggin B/ChrIX* gene (Fig. 3A).

There were no interchromosomal chimeric spiggin genes observed in the 2006 draft (Broad/gasAcu1) assembly of *G. aculeatus*. This may reflect errors in the draft assembly of the *G. aculeatus* genome (Roesti *et al*. 2013), as draft assemblies are often incorrect in annotating multigene family copy number (Denton *et al*. 2014) and whole‐genome shotgun assemblies are typically poor at adequately resolving repeat structures (She *et al*. 2004). Alternatively, retrotransposition of spiggin genes may not have occurred in the sequenced individual, which was selected from an inbred laboratory population exhibiting a low level of genetic heterogeneity (Kingsley & Peichel 2007).

In contrast to retrotransposition, unequal crossing over tends to generate tandem intronic gene duplication on the same chromosome (Zhang 2003). The genome assembly of *G. aculeatus* shows six spiggin genes arranged in tandem on chromosome IV (Fig. 5), and so it is plausible that these spiggin genes were generated through unequal crossing over. In this study, ten intrachromosomal chimeric transcripts were sequenced, each consisting of exons from two different spiggin genes. A further five intrachromosomal spiggin chimerics were identified from the public domain. Although it is not known where or how these chimerics are arranged in the genomes of the individuals from which they were obtained, these transcripts all contain exons from different spiggin genes that are tandemly arranged on chromosome IV of the draft assembly, and so it seems likely that reciprocal unequal crossing over and unidirectional gene conversion played a part in their generation. Additionally, partial sequencing of *spiggin B/C1* and *spiggin B/C2* chimeric genes from genomic DNA showed the presence of introns, a feature of duplication by unequal crossing over (Zhang 2003), rather than retrotransposition (Figs 3B and S1, Supporting information). Finally, one of the six spiggin loci on the draft *G. aculeatus* genome, *spiggin B3* (spg4/ENSGACT00000025255), was identified as an intrachromosomal chimeric of *spiggin B1* (ENSGACT00000025226) and *spiggin B2* (ENSGACT00000025256).

### Analysis of the *G. aculeatus* draft genome assembly

As L1 retrotransposons have been shown to serve as hotspots for unequal crossing over (Burwinkel & Kilimann 1998; Cordaux & Batzer 2009; Finnegan 2012), the same L1 elements that likely caused retrotransposition may have also been responsible for generating all the initial spiggin gene duplications that are tandemly arranged on chromosome IV. Our analysis of the *G. aculeatus* draft assembly revealed 10 partial L1 retrotransposon sequences in the 219‐kb region of chromosome IV (Chr:group IV 21002172–21221912) known to contain all annotated spiggin genes (Kawahara & Nishida 2007). Originally much longer, at 5–7 kb (Vandergon & Reitman 1994), these L1 retrotransposons have been truncated at the 5’ end to between 439 bp and 1512 bp in length (Fig. 5). The 99% homology between two of the Chicken repeat 1–3 (CR1‐3) L1 retrotransposons as well as the 92% homology between 3 of the CR1‐1L1 retrotransposons may have provided hotspots of ectopic sequence similarity for unequal crossing over (Fig. 5).

### Spiggin protein predictions

The in silico translations of the nonchimeric *spiggin B*,* spiggin C1* and *spiggin C2* putative ORFs sequenced from *G. aculeatus* predicted significant differences at the protein level. Spiggin B was almost three times the length of spiggin C1 and spiggin C2, and although all three spiggin proteins contained vWD and C8 doma‐ins, the numbers of each domain differed between proteins. Conserved domain searches also revealed a trypsin inhibitor‐like cysteine‐rich (TIL) domain in spiggin C1 and spiggin C2, but not in spiggin B. Peptides containing TIL domains are known to be antimicrob‐ial (Zeng *et al*. 2013), so the expression of spiggin genes C1 and C2 could confer the known antimicrobial properties of spiggin (Little *et al*. 2008). It was shown that of the three spiggins, only spiggin B had dimerization and multimerization motifs, features typical of mucin proteins (Perez‐Vilar & Hill 1998). Spiggin C1 and spiggin C2 each had a truncated dimerization motif, and although this truncated motif is conserved in other secretory proteins (Gum *et al*. 1994; Joba & Hoffmann 1997), the lack of both types of motif suggests spiggin C1 and spiggin C2 proteins are secreted as monomers.

All mucin proteins undergo the process of glycosylation in which carbohydrates (glycans) are attached to the protein. Whilst in the endoplasmic reticulum and before dimerization, mucins are N‐linked glycosylated (Perez‐Vilar & Hill 1999). Although the numbers of N‐linked glycosylation sites are similar between spiggins B, C1 and C2, their locations differ, which suggests differences in protein structure or function (Imperiali & O'Connor 1999). Following N‐linked glycosylation, oligosaccharide side chains are attached to the mucins via O‐linked glycosylation in the Golgi apparatus (Perez‐Vilar & Mabolo 2007). These oligosaccharides allow the hydration of mucin and contribute to gel formation (Bansil *et al*. 1995), but there are significant differences in the number of O‐linked glycosylation sites between spiggin proteins. Typical for a mucin protein, the spiggin B proteins have a high number of predicted O‐linked glycosylation sites, while spiggins C1 and C2 have nearly 10‐fold fewer. No full‐length *spiggin A* transcripts were sequenced in this study, but in silico translation of the longest alternatively spliced *spiggin A* transcript predicted a relatively high number of nine O‐linked glycosylation sites for the 1087 bp length and a multimerisation motif in the vWD domain. These observations indicate that *spiggin A* shows greater similarity to the mucin‐like *spiggin B* gene than to either *spiggin C1* or *spiggin C2*.

The *G. aculeatus* intrachromosomal spiggin chimerics were shown to differ from their parental spiggins in the number and location of predicted N‐linked and O‐linked glycosylation sites, suggesting different structures and/or properties (Fig. 6, translations 3–5). In two of the four interchromosomal chimerics, the short spiggin region was predicted to contain a vWD domain with a full multimerization motif, but with no O‐linked glycosylation sites (Fig. 6, translations 6 and 7). The above differences in protein motifs and level of post‐translational glycosylation are likely to result in significantly different properties or functions between spiggin proteins, which through differential spiggin gene expression could allow for the production of different forms of nesting glue. A recent study showed that of *spiggin B*,* C1* and *C2*, only the mucin‐like *spiggin B* was significantly up‐regulated in the kidneys of male *G. aculeatus* constructing nests in flowing water compared to still water conditions (Seear *et al*. 2014). These findings support the hypothesis that the differential expression of various spiggin genes might generate nesting glues with different functional properties, suggesting that individual male fish can plastically adjust not only the quantity but also the structural properties of glue in response to environmental change.

## Concluding remarks

The characterization of the spiggin multigene family of *Gasterosteus aculeatus* resolved three main lineages (A, B and C) and further subdivisions of B (B1, B2) and C (C1, C2). Our analysis also revealed that *spiggin C1* and *spiggin C2* genes have diverged substantially from *spiggin A* and *spiggin B1/B2*. In silico translations indicate that while spiggin B has mucin‐like features, spiggins C1 and C2 are secreted as short – possibly antimicrobial – monomers. Similar to the conventional view pioneered by Ohno (1970), we propose that the duplication of *spiggin B* has freed the duplicate (*spiggin C*) from purifying selection and that subsequent mutations have allowed initial divergence and the evolution of new functions. Our discovery of 22 chimeric *G. aculeatus* spiggin genes from a wide range of populations sampled in this and other studies (Jones *et al*. 2001; Kawasaki *et al*. 2003; Kawahara & Nishida 2006) suggests that further gene duplication and diversification has occurred separately in different populations, through unequal crossing over, gene conversion and retrotransposition (Fig. 1B).

As spiggin is secreted in the external environment, it is exposed to a wide range of nonbuffered aquatic conditions, so local adaptation of this protein is predic‐ted (Kawahara & Nishida 2007). This hypothesis is supported by Roesti *et al*. (2014), who found genomic evidence for divergent selection between marine and freshwater populations of *G. aculeatus* at the spiggin multigene cluster. The diversification of *spiggin C1* and *spiggin C2* genes from the *spiggin B* duplications, along with subsequent chimeric gene generation, may have allowed sticklebacks to produce nesting glues with different functional properties. Indeed, it has been demonstrated that while *spiggin B* was up‐regulated in nesting *G. aculeatus* due to an increase in flow rate, *spiggin C1* and *spiggin C2* were not (Seear *et al*. 2014). This gene diversity is consistent with the hypothesis of local adaptation of the spiggin protein to diverse freshwater habitat types following their colonization by marine stickleback populations (Roesti *et al*. 2014).

**Figure 7:**
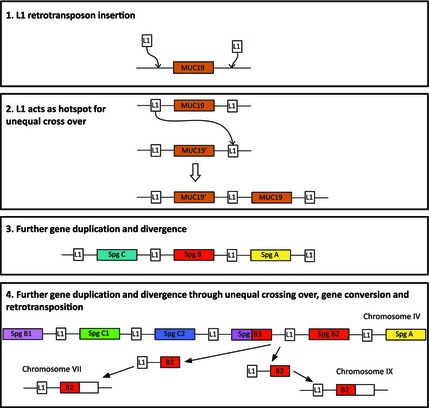
Proposed model of spiggin gene amplification by L1 retrotransposon‐mediated unequal crossing over, gene conversion and retrotransposition. Boxes 1 and 2 show how insertion of L1 retrotransposons on either side of the ancestral single‐copy *MUC19* may have been responsible for the initial gene duplication through unequal crossing over. Box 3 indicates how further gene duplication and divergence could have led to the three major spiggin gene lineages, A, B and C. Box 4 indicates how retrotransposition has led to further gene duplication.

Finally, the sequencing and comparative analysis of spiggin genes from *G. aculeatus* and *Pungitius pungitius*, including intrachromosomal and interchromosomal chimeric spiggin genes from both species, provides strong support for the hypothesis that L1 retrotransposons have been responsible for the successive duplication of an ancestral single‐copy *MUC19* gene into a spiggin multigene family (Fig. 7), which has subsequently allowed sticklebacks to produce copious glue protein for nest construction. We propose that insertion of L1 retrotransposons near the ancestral *MUC19* gene created recombination hotspots leading to tandem gene duplication through unequal crossing over. Spiggin duplicates freed from purifying selection diversified through mutations, before subsequent L1 retrotransposition, unequal crossing over and gene conversion events resulted in spiggin interchromosomal and intrachromosomal chimerics.

P.J.S., W.P.G.C., E.R. and I.B. contributed to the conceptual development and design of the study and to the interpretation of results; fieldwork and aquarium studies were undertaken by I.B. and P.J.S. The laboratory work was coordinated by P.J.S. who also isolated the spiggin genes, processed and aligned sequences, and drafted the manuscript; W.P.G.C. performed sequence alignments and phylogenetic analyses. All authors read, commented on and approved the final manuscript.

## Data accessibility

All spiggin sequences in this study including genomic DNA sequences, cDNA transcripts and M13 forward and reverse sequences have been submitted to the DDBJ/EMBL/GenBank databases under Accession nos AB909965–AB910047, AB936833‐AB936834, JZ555463‐JZ555920, JZ583854‐JZ584097 and dbEST: 78920304–78920761, 79255600–79255843. All sequence alignments are available from the Dryad Digital Repository: http://dx.doi.org/10.5061/dryad.pc5n9.

## Supporting information


**Table S1** Primers used for cloning and sequencing.
**Table S2** List of full‐length spiggin cDNA transcripts.
**Table S3** List of spiggin gDNA sequences.
**Fig. S1** Verification of intrachromosomal spiggin chimerics using internal reverse primers.
**Fig. S2** PCR of *spiggin B/ChrIX* interchromosomal gene from genomic DNA of three marine (SAL) and three freshwater (EDH) *G. aculeatus*.
