# Supplementary material for: The molecular evolution of spiggin nesting glue in sticklebacks
Source: Mol Ecol. 2015 Aug 3;24(17):4474–88. doi: 10.1111/mec.13317 (PMC4989455; doi:10.1111/mec.13317)
Supplement: Supplementary file 1 — Table S1 Primers used for cloning and sequencing. Table S2 List of full‐length spiggin cDNA transcripts. Table S3 List of spiggin gDNA sequences. Fig. S1 Verification of intrachromosomal spiggin chimerics using internal reverse primers. Fig. S2 PCR of spiggin B/ChrIX interchromosomal gene from genomic DNA of three marine (SAL) and three freshwater (EDH) G. aculeatus. [file MEC-24-4474-s001.docx]

**Supporting information**

Table S1. Primers used for cloning and sequencing.

| **Primer name** | **5´-3´ sequence** | **Purpose in study** | **Source** |
| --- | --- | --- | --- |
| SPG5R01 | GCTTGCTGTCTCTGATCAACTCGTC | 5´ RACE PCR | Kawahara & Nishida 2006 |
| SPG5R04 | GACGCTCTGGTTGTTTTCAAAGCC | 5´ RACE PCR | Kawahara & Nishida 2006 |
| SPG5A1C1 | TTTCCCACCATCACACGTACATGACT | 5´ RACE PCR | GenBank:JK993477-JK993535 |
| SPG5F1 | CTGCTCTCCTGCAGCATCAGGCA | Full-length PCR | This study |
| Spg 1A alpha F1 | CGAGGTGTCCAGGAGACCTGAT | Sequencing | This study |
| Spg alpha F2 | GCACCAACCTGGAGAATGAGAA | Sequencing | This study |
| Alpha 2538F | TGTGACATGAGAAGAGGCACC | Sequencing | This study |
| Alpha 3322F | ATCTCTCACAAGCTGGAAGGC | Sequencing | This study |
| Alpha 500R | GTCCAGCACCACACATAGAGC | Sequencing | This study |
| Alpha 5019F | AGTCCGGCTGTCACATTTGTA | Sequencing | This study |
| Spg type2 F1 | GACTCATAAGCTTCACTGTCAACCAGAA | Sequencing | This study |
| Spg type2 F3 | CTAGGTGTCCAGGAGATCTGATT | Sequencing | This study |
| Spg alpha R1 | ATCTGGCCGGATGACAATGGACT | Sequencing | This study |
| Spg type1A R2 | TTGGTTGGTTCAACACTGCACAC | Sequencing | This study |
| Spg type 2 R3 | TCGGAAAGAACCCGGTTTC | Sequencing | This study |
| ChrIX R | GACAGGTGAAACACACACAAATAC | Standard PCR | This study |
| Spg alpha F | TGAAAACCAAGAACTGTCTGCAAG | Standard PCR | This study |
| Spg alpha1R | GTCGTATTTCTCCGTTTCTGTAAC | Standard PCR | This study |
| Spg C1 R3 | TGCTGGACCCTTTTCCCTATAT | Standard PCR | This study |
| Spg C2 R2 | TGGACCATTTTCCCGATAAATCAGA | Standard PCR | This study |

Table S2. List of full-length spiggin cDNA transcripts.

| **Accession number** | **DDBJ/EMBL/GenBank ID** | **Description** | **Length**  **(bp)** | **Stickleback location** |
| --- | --- | --- | --- | --- |
|  | ENSGACT00000025258 | Spiggin A | 2562 | Bear Paw Lake, Alaska (Jones *et al.* 2012) |
|  | ENSGACT00000025226 | Spiggin B1 | 3556 | Bear Paw Lake, Alaska (Jones *et al.* 2012) |
|  | ENSGACT00000025256 | Spiggin B2 | 2718 | Bear Paw Lake, Alaska (Jones *et al.* 2012) |
|  | ENSGACT00000025255 | Spiggin B3 | 3174 | Bear Paw Lake, Alaska (Jones *et al.* 2012) |
|  | ENSGACT00000025243 | Spiggin C1 | 1857 | Bear Paw Lake, Alaska (Jones *et al.* 2012) |
|  | ENSGACT00000025250 | Spiggin C2 | 1911 | Bear Paw Lake, Alaska (Jones *et al.* 2012) |
| AB243101 | Spiggin type-1A | Spiggin C1 | 2253 | Dorset, UK (Kawasaki *et al.* 2003) |
| AB243102 | Spiggin type-1B | Chimeric: 2251bp Spiggin C1/646 bp DW655150 on Chromosome III | 2897 | Dorset, UK (Kawasaki *et al.* 2003) |
| AB243103 | Spiggin type-1C | Chimeric: 2250bp Spiggin C1/1542bp spiggin B | 3792 | Dorset, UK (Kawasaki *et al.* 2003) |
| AB243104 | Spiggin type-2 | Spiggin C2 | 2261 | Dorset, UK (Kawasaki *et al.* 2003) |
| AF323732 | Spiggin alpha | Chimeric: 2729bp Spiggin B2/1439bp DN653172 on Chromosome I | 4168 | Baltic Sea, Sweden (Jones *et al.* 2001) |
| AF323733 | Spiggin beta | Alternatively spliced spiggin B | 2179 | Baltic Sea, Sweden (Jones *et al.* 2001) |
| AF323734 | Spiggin gamma | Chimeric: 1409bp Spiggin B/48bp BT026625 on Chromosome X | 1457 | Baltic Sea, Sweden (Jones *et al.* 2001) |
| AB221477 | Spiggin 1.1 | Alternatively spliced spiggin B | 3734 | Eastern Hokkaido, Japan (Kawahara & Nishida 2006) |
| AB221478 | Spiggin 1.2 | Alternatively spliced spiggin B | 2860 | Eastern Hokkaido, Japan (Kawahara & Nishida 2006) |
| AB221479 | Spiggin 1.3 | Alternatively spliced spiggin B | 2745 | Eastern Hokkaido, Japan (Kawahara & Nishida 2006) |
| AB221480 | Spiggin 1.4 | Chimeric: 1851bp Spiggin B/401bp Spiggin C1 | 2252 | Eastern Hokkaido, Japan (Kawahara & Nishida 2006) |
| AB221481 | Spiggin 2 | Chimeric: 1720bp Spiggin C2/1357bp Spiggin A | 3077 | Eastern Hokkaido, Japan (Kawahara & Nishida 2006) |
| AB221482 | Spiggin 3 | Chimeric: 1572bp Spiggin C2/1505bp Spiggin A | 3077 | Eastern Hokkaido, Japan (Kawahara & Nishida 2006) |
| AB221483 | Spiggin 4 | Chimeric: 1565bp Spiggin B/198bp Spiggin A/1824bp Spiggin B | 3587 | Eastern Hokkaido, Japan (Kawahara & Nishida 2006) |
| AB910010 | Spiggin B | Spiggin B | 5787 | Sälvik, Sweden (SAL01) |
| AB910011 | Spiggin B | Spiggin B | 5788 | Sälvik, Sweden (SAL02) |
| AB910012 | Spiggin B | Spiggin B | 5837 | Edinburgh, UK (EDH07) |
| AB910013 | Spiggin C1 | Spiggin C1 | 2265 | Edinburgh, UK (EDH01) |
| AB910014 | Spiggin C1 | Spiggin C1 | 2265 | Edinburgh, UK (EDH05) |
| AB910015 | Spiggin C1 | Spiggin C1 | 2266 | Sälvik, Sweden (SAL03) |
| AB910016 | Spiggin C1 | Spiggin C1 | 2269 | Sälvik, Sweden (SAL01) |
| AB910017 | Spiggin C1 | Spiggin C1 | 2270 | Edinburgh, UK (EDH07) |
| AB910018 | Spiggin C1 | Spiggin C1 | 2271 | Edinburgh, UK (EDH01) |
| AB910019 | Spiggin C1 | Spiggin C1 | 2271 | Sälvik, Sweden (SAL03) |
| AB910020 | Spiggin C1 | Spiggin C1 | 2272 | Edinburgh, UK (EDH05) |
| AB910021 | Spiggin C1 | Spiggin C1 | 2272 | Sälvik, Sweden (SAL01) |
| AB910022 | Spiggin C2 | Spiggin C2 | 2273 | Edinburgh, UK (EDH01) |
| AB910023 | Spiggin C2 | Spiggin C2 | 2274 | Edinburgh, UK (EDH07) |
| AB910024 | Spiggin C2 | Spiggin C2 | 2275 | Edinburgh, UK (EDH01) |
| AB910025 | Spiggin C2 | Spiggin C2 | 2275 | Sälvik, Sweden (SAL03) |
| AB910026 | Spiggin C2 | Spiggin C2 | 2276 | Edinburgh, UK (EDH05) |
| AB910000 | Spiggin A/B chimeric | 1172bp Spiggin B/257bp Spiggin A/4362bp Spiggin B | 5791 | Edinburgh, UK (EDH05) |
| AB910001 | Spiggin B/C chimeric | 779bp Spiggin B/1489bp C1 | 2268 | Sälvik, Sweden (SAL01) |
| AB910002 | Spiggin B/C chimeric | 291bp Spiggin C1/52bp Spiggin B/1973bp Spiggin C1 | 2316 | Sälvik, Sweden (SAL01) |
| AB910003 | Spiggin C chimeric | 1880bp Spiggin C1/325bp Spiggin C2 | 2205 | Edinburgh, UK (EDH05) |
| AB910004 | Spiggin C chimeric | 1880bp Spiggin C1/325bp Spiggin C2 | 2205 | Edinburgh, UK (EDH05) |
| AB910005 | Spiggin C chimeric | 429bp Spiggin C1/1784bp Spiggin C2 | 2213 | Sälvik, Sweden (SAL01) |
| AB910006 | Spiggin C chimeric | 779bp Spiggin C1/1431bp Spiggin C2 | 2210 | Edinburgh, UK (EDH05) |
| AB910007 | Spiggin C chimeric | 343bp Spiggin C1/253bp Spiggin C2/1737bp Spiggin C1 | 2333 | Sälvik, Sweden (SAL03) |
| AB910008 | Spiggin C chimeric | 343bp Spiggin C2/1927bp Spiggin C1 | 2270 | Sälvik, Sweden (SAL03) |
| AB910009 | Spiggin C chimeric | 556bp Spiggin C2/1773bp Spiggin C1 | 2329 | Edinburgh, UK (EDH05) |
| AB910027 | Spiggin B/Chr I retrogene | Chimeric: 119bp Spiggin B/728bp EIF4H on Chromosome I | 847 | Sälvik, Sweden (SAL02) |
| AB910028 | Spiggin B/Chr IX retrogene | Chimeric: 208bp Spiggin B/207bp BT26706 on Chromosome IX | 415 | Sälvik, Sweden (SAL01) |
| AB910029 | Spiggin B/Chr V retrogene | Chimeric: 606 bp Spiggin B/153bp SULT1ST6 on Chromosome V | 759 | Sälvik, Sweden (SAL01) |
| AB910030 | Spiggin B/Chr VII retrogene | Chimeric: 691bp Spiggin B/363bp Chromosome VII | 1054 | Sälvik, Sweden (SAL02) |
| AB909995 | Spiggin B/A chimeric alternatively spliced variant 1 | Chimeric: 294 bp Spiggin B/789bp Spiggin A | 1083 | Sälvik, Sweden (SAL02) |
| AB909996 | Spiggin B/A chimeric alt spliced variant 2 | Chimeric: 294 bp Spiggin B/789bp Spiggin A | 1090 | Sälvik, Sweden (SAL02) |
| AB909997 | Spiggin B/A chimeric alt spliced variant 3 | Chimeric: 294 bp Spiggin B/789bp Spiggin A | 497 | Sälvik, Sweden (SAL02) |
| AB909998 | Spiggin B/C chimeric alt spliced | Chimeric: 401bp Spiggin B/1751bp Spiggin C2 | 2152 | Edinburgh, UK (EDH01) |
| AB909999 | Spiggin C/A chimeric alt spliced | Chimeric: 793bp Spiggin C1/270bp Spiggin A | 1063 | Sälvik, Sweden (SAL01) |
| AB910033 | Spiggin C/B chimeric alt spliced | Chimeric: 59 bp Spiggin C2/190bp Spiggin B | 249 | Sälvik, Sweden (SAL01) |
| AB909965 | Spiggin A alternatively spliced variant 1 | Alternatively spliced Spiggin A | 371 | Edinburgh, UK (EDH07) |
| AB909966 | Spiggin A alternatively spliced variant 2 | Alternatively spliced Spiggin A | 375 | Sälvik, Sweden (SAL02) |
| AB909967 | Spiggin A alternatively spliced variant 3 | Alternatively spliced Spiggin A | 380 | Sälvik, Sweden (SAL02) |
| AB909968 | Spiggin A alternatively spliced variant 4 | Alternatively spliced Spiggin A | 1077 | Sälvik, Sweden (SAL02) |
| AB909969 | Spiggin A alternatively spliced variant 5 | Alternatively spliced Spiggin A | 1087 | Sälvik, Sweden (SAL02) |
| AB910035 | Spiggin A alternatively spliced variant 6 | Alternatively spliced Spiggin A | 133 | Sälvik, Sweden (SAL01) |
| AB909970 | Spiggin B alternatively spliced variant 1 | Alternatively spliced Spiggin B | 513 | Sälvik, Sweden (SAL02) |
| AB909971 | Spiggin B alternatively spliced variant 2 | Alternatively spliced Spiggin B | 1100 | Sälvik, Sweden (SAL02) |
| AB909972 | Spiggin B alternatively spliced variant 3 | Alternatively spliced Spiggin B | 1107 | Sälvik, Sweden (SAL02) |
| AB909973 | Spiggin B alternatively spliced variant 4 | Alternatively spliced Spiggin B | 168 | Sälvik, Sweden (SAL02) |
| AB909974 | Spiggin B alternatively spliced variant 5 | Alternatively spliced Spiggin B | 786 | Sälvik, Sweden (SAL02) |
| AB909975 | Spiggin B alternatively spliced variant 6 | Alternatively spliced Spiggin B | 672 | Sälvik, Sweden (SAL02) |
| AB909976 | Spiggin B alternatively spliced variant 7 | Alternatively spliced Spiggin B | 673 | Sälvik, Sweden (SAL02) |
| AB909977 | Spiggin B alternatively spliced variant 8 | Alternatively spliced Spiggin B | 577 | Sälvik, Sweden (SAL02) |
| AB909978 | Spiggin B alternatively spliced variant 9 | Alternatively spliced Spiggin B | 729 | Sälvik, Sweden (SAL02) |
| AB909979 | Spiggin B alternatively spliced variant 10 | Alternatively spliced Spiggin B | 734 | Sälvik, Sweden (SAL02) |
| AB909980 | Spiggin B alternatively spliced variant 11 | Alternatively spliced Spiggin B | 743 | Sälvik, Sweden (SAL01) |
| AB910036 | Spiggin B alternatively spliced variant 12 | Alternatively spliced Spiggin B | 120 | Sälvik, Sweden (SAL01) |
| AB910034 | Spiggin B alternatively spliced variant 13 | Alternatively spliced Spiggin B | 124 | Sälvik, Sweden (SAL01) |
| AB910038 | Spiggin B alternatively spliced variant 14 | Alternatively spliced Spiggin B | 138 | Sälvik, Sweden (SAL02) |
| AB910039 | Spiggin B alternatively spliced variant 15 | Alternatively spliced Spiggin B | 848 | Sälvik, Sweden (SAL02) |
| AB910032 | Spiggin B alternatively spliced variant 16 | Alternatively spliced Spiggin B | 850 | Sälvik, Sweden (SAL01) |
| AB910037 | Spiggin B alternatively spliced variant 17 | Alternatively spliced Spiggin B | 857 | Sälvik, Sweden (SAL01) |
| AB909981 | Spiggin C1 alternatively spliced variant 1 | Alternatively spliced Spiggin C1 | 973 | Sälvik, Sweden (SAL01) |
| AB909982 | Spiggin C1 alternatively spliced variant 2 | Alternatively spliced Spiggin C1 | 975 | Sälvik, Sweden (SAL01) |
| AB909983 | Spiggin C1 alternatively spliced variant 3 | Alternatively spliced Spiggin C1 | 2314 | Edinburgh, UK (EDH07) |
| AB909984 | Spiggin C1 alternatively spliced variant 4 | Alternatively spliced Spiggin C1 | 2316 | Edinburgh, UK (EDH05) |
| AB909985 | Spiggin C1 alternatively spliced variant 5 | Alternatively spliced Spiggin C1 | 2316 | Edinburgh, UK (EDH05) |
| AB909986 | Spiggin C1 alternatively spliced variant 6 | Alternatively spliced Spiggin C1 | 2238 | Sälvik, Sweden (SAL01) |
| AB909987 | Spiggin C1 alternatively spliced variant 7 | Alternatively spliced Spiggin C1 | 2314 | Sälvik, Sweden (SAL03) |
| AB909988 | Spiggin C1 alternatively spliced variant 8 | Alternatively spliced Spiggin C1 | 2182 | Edinburgh, UK (EDH07) |
| AB909989 | Spiggin C1 alternatively spliced variant 9 | Alternatively spliced Spiggin C1 | 950 | Edinburgh, UK (EDH01) |
| AB910031 | Spiggin C1 alternatively spliced variant 10 | Alternatively spliced Spiggin C1 | 127 | Edinburgh, UK (EDH01) |
| AB909990 | Spiggin C2 alternatively spliced variant 1 | Alternatively spliced Spiggin C2 | 740 | Sälvik, Sweden (SAL01) |
| AB909991 | Spiggin C2 alternatively spliced variant 2 | Alternatively spliced Spiggin C2 | 895 | Sälvik, Sweden (SAL01) |
| AB909992 | Spiggin C2 alternatively spliced variant 3 | Alternatively spliced Spiggin C2 | 2129 | Sälvik, Sweden (SAL01) |
| AB909993 | Spiggin C2 alternatively spliced variant 4 | Alternatively spliced Spiggin C2 | 138 | Sälvik, Sweden (SAL01) |
| AB909994 | Spiggin C2 alternatively spliced variant 5 | Alternatively spliced Spiggin C2 | 2209 | Edinburgh, UK (EDH05) |
| AB936833 | Spiggin/ChrI retrogene | Chimeric: 230bp Spiggin/77bp *P. pungitius* unique insertion/641bp *G. aculeatus* Chromosome I | 948 | River Welland, UK |
| AB936834 | Spiggin/ChrXIII retrogene | Chimeric: 266bp Spiggin/113bp *P. pungitius* unique insertion/1108bp *G. aculeatus* Chromosome I | 1487 | River Welland, UK |
| XM_006781117 | PREDICTED: *Neolamprologus brichardi* mucin-19-like mRNA | Spiggin outgroup | 5496 |  |

Table S3. List of spiggin gDNA sequences.

| **Accession number** | **Genbank name** | **Length (bp)** | **Stickleback location** |
| --- | --- | --- | --- |
| AB910040 | Spiggin B/C2_gDNA_CAR45_2 | 600 | Carsington Reservoir, UK |
| AB910041 | Spiggin B/C1_gDNA_CAR45_4 | 569 | Carsington Reservoir, UK |
| AB910042 | Spiggin B/C1_gDNA_CAR45_6 | 600 | Carsington Reservoir, UK |
| AB910043 | Spiggin B/C2_gDNA_CAR45_7 | 583 | Carsington Reservoir, UK |
| AB910044 | Spiggin B/ChrIX retrogene_gDNA_EDH07_2 | 370 | Edinburgh, UK |
| AB910045 | Spiggin B/ChrIX retrogene_gDNA_SAL02_9 | 370 | Sälvik, Sweden |
| AB910046 | Spiggin B/C1_gDNA_WEL06_1 | 611 | River Welland, UK |
| AB910047 | Spiggin B/C1_gDNA_WEL06_5 | 595 | River Welland, UK |

**SpgB**

**WEL02 gDNA WEL06 gDNA CAR44 gDNA**

PCR1 PCR2 PCR1 PCR2 PCR1 PCR2


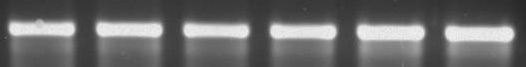


**SpgB/C1**

**WEL02 gDNA WEL06 gDNA CAR44 gDNA WEL03 SpgB/C1**

**1kb ladder** **Water** PCR1 PCR2 PCR1 PCR2 PCR1 PCR2  **cDNA gDNA clone**


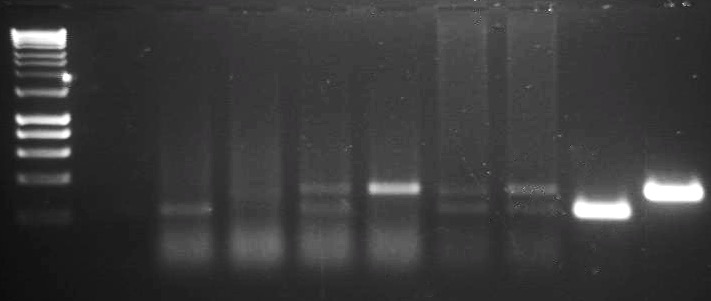


**SpgB/C2**

**WEL02 gDNA WEL06 gDNA CAR44 gDNA WEL03 SpgB/C2**

**1kb ladder** **Water** PCR1 PCR2 PCR1 PCR2 PCR1 PCR2 **cDNA gDNA clone**

**
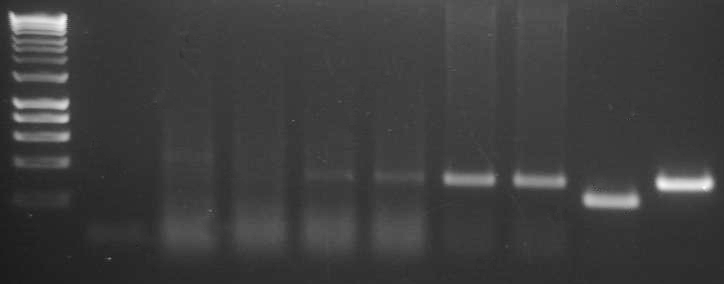
**

**Fig. S1. Verification of intrachromosomal spiggin chimerics using internal reverse primers.**

Independent replicate PCRs (for details, see Materials and Methods) of *SpgB/C1*, *SpgB/C2* and the non-chimeric control, *SpgB* were performed with genomic DNA from three freshwater *G. aculeatus* individuals (WEL = River Welland, UK; CAR = Carsington Reservior, UK). Negative controls consisted of water (instead of template) and cDNA (from WEL03) to show a smaller size PCR product than that generated from gDNA. The positive control consisted of the original genomic DNA chimeric clones generated with Spg alpha F and Spg alpha1R primers as described in the Materials and Methods. PCR products were electrophoresed on 2% (w/v) agarose gels. The arrows indicate the expected PCR product size from genomic DNA (325bp for *SpgB/C1* and 318bp for *SpgB/C2*). These PCRs show that the spiggin chimeric genes *SpgB/C1* and *SpgB/C2* are present in the genomic DNA of WEL06 and CAR44, but not in WEL02, demonstrating the variation in spiggin genes between *G. aculeatus* individuals.

**SpgB/ChrIX**

**1kb ladder SAL01 SAL02 SAL03 EDH01 EDH05 EDH07 H_2_O**


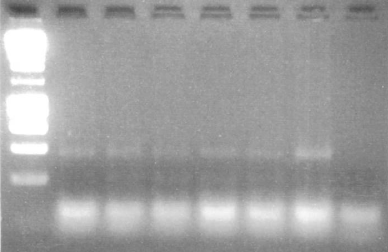


Spg/ChrIX (382bp)

**Fig. S2. PCR of *spiggin B/ChrIX* interchromosomal gene from genomic DNA of three marine (SAL) and three freshwater (EDH) *G. aculeatus*.** The arrow indicates the expected size for the intronless interchromosomal spiggin gene. A water negative control was used in the PCR to rule out cDNA contamination.
